# Why Current Doppler Ultrasound Methodology Is Inaccurate in Assessing Cerebral Venous Return: The Alternative of the Ultrasonic Jugular Venous Pulse

**DOI:** 10.1155/2016/7082856

**Published:** 2016-02-24

**Authors:** Paolo Zamboni

**Affiliations:** Vascular Diseases Center, University of Ferrara, Via Aldo Moro 8, Cona, 44124 Ferrara, Italy

## Abstract

Assessment of cerebral venous return is growing interest for potential application in clinical practice. Doppler ultrasound (DUS) was used as a screening tool. However, three meta-analyses of qualitative DUS protocol demonstrate a big heterogeneity among studies. In an attempt to improve accuracy, several authors alternatively measured the flow rate, based on the product of the time average velocity with the cross-sectional area (CSA). However, also the quantification protocols lacked of the necessary accuracy. The reasons are as follows: (a) automatic measurement of the CSA assimilates the jugular to a circle, while it is elliptical; (b) the use of just a single CSA value in a pulsatile vessel is inaccurate; (c) time average velocity assessment can be applied only in laminar flow. Finally, the tutorial describes alternative ultrasound calculation of flow based on the Womersley method, which takes into account the variation of the jugular CSA overtime. In the near future, it will be possible to synchronize the electrocardiogram with the brain inflow (carotid distension wave) and with the outflow (jugular venous pulse) in order to nicely have a noninvasive ultrasound picture of the brain-heart axis. US jugular venous pulse may have potential use in neurovascular, neurocognitive, neurosensorial, and neurodegenerative disorders.

## 1. Doppler Ultrasound Assessment of Cerebral Venous Return

In the last years, there was a growing interest in understanding the role of brain drainage in neurovascular and in neurodegenerative disorders. The description of chronic cerebrospinal venous insufficiency (CCSVI) leads numerous researchers to explore the role of restricted venous outflow in favouring multiple sclerosis (MS) [1–5], Alzheimer's disease [6], Parkinson's disease [7], Meniere disease [8], and migraine [9–11]. From a behavioural point of view, attention was also given to sleeping disorders [12] and to chronic fatigue and cognitive features [13, 14], always in relationship with CCSVI.

Since CCSVI straights the extracranial major veins of the neck, ultrasound echo color-Doppler (ECD) was chosen as a screening methodology, either because of the noninvasiveness or because it is widespread equipment. The screening was initially proposed through a qualitative investigation based on a list of five physiological criteria; subsequently, they were very well detailed in a consensus conference [4, 15]. The reproducibility of the ECD protocol was tested by some authors [16, 17]. However, despite the good inter- and intravariability coefficients obtained among expert operators, ECD screening gave highly variable results in the published studies. Particularly, three meta-analyses investigated the prevalence of CCSVI in MS, and all agreed with the higher prevalence of CCSVI in MS [18–20]. However, according to such meta-analysis the variability among studies is really too much elevated for considering ECD a good screening tool. This conclusion feeds a scientific controversy between confirmatory and not confirmatory investigators [21, 22].

## 2. Methodology for ECD Venous Flow Assessment

To overcome the controversy, the ISNVD published a position paper which substantially proposes a multimodality diagnostic protocol for the diagnosis of CCSVI. The article discusses the advantages and the limits of the different techniques which can be used to assess cerebral venous return [23]. According to this document, ultrasound needs to be necessarily complemented by MRV, plethysmography on the noninvasive side, and catheter venography and intravascular ultrasound on the invasive one.

However, ultrasound remains an ideal technique for screening, and several investigators tried to measure the jugular and vertebral vein flow by the means of ECD in order to substitute the operator dependent qualitative protocol with a more objective measurement of the venous flow [24–29].

All the ECD equipment on the market allows applying the Poiseuille law for the assessment of flow:

(1) $$\begin{array}{c} Q=v\times CSA, \end{array}$$

where *Q* is the flow rate, *v* is the velocity expressed as time average velocity (TAV), and CSA is the cross-sectional area of the vessel under investigation.

**Figure 1:**
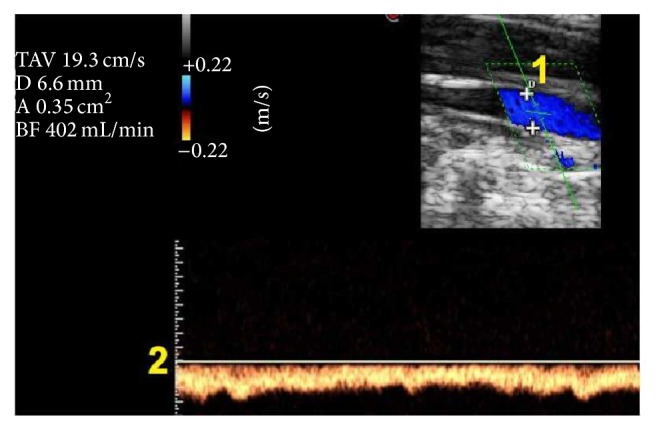
Current ECD assessment of flow rate in the internal jugular vein. The flow is automatically calculated by the product of the diameter (1) with the TAV (2). In this case, the TAV, visible on the left upper side of the figure, is 19.3 cm/sec. The diameter (D) is 6.6 mm. With the arbitrary assumption of the circular shape of the vein, the area of the vein (A) is 0.35 cm^2^. Consequently the flow rate is 402 mL/min.

Practically the available ECD instruments permit measuring the flow automatically by the combination of the B-mode imaging with the Doppler spectra analysis. In Figure 1, we give an example of the measure of flow through the product of TAV with CSA.

Thanks to this technological possibility, some authors assessed the presence of CCSVI in healthy people and in neurodegenerative diseases, but again obtaining variable results [24–29].

## 3. Comparison among Ultrasound Studies Assessing Cerebral Outflow

Flow quantification was always assessed, as anticipated above, by applying the formula of Poiseuille. Valdueza et al. [24] measuring the flow rate in the jugular and in the vertebral veins, respectively, in supine position and in upright position found that the latter was the predominant outflow route in upright position. In normal people, they measured at 0° a flow of 700 mL/min (270) in the internal jugular veins (IJVs) and another of 40 mL/min (20) in the vertebral veins (VVs); with the head at 90°, the flow rate changed completely and was 70 mL/min (100) and 210 mL/min (120), respectively, in the IJVs and in the VVs. The authors admitted a lack of about 450 mL/min when the subject was standing despite the increased flow in the VVs, by comparing cerebral flow in supine versus upright postures. This result should be evaluated looking also at the change in CSA. The author measured in the IJV 106 mm^2^ in supine versus 17 mm^2^ in upright, respectively.

The observation of Valdueza was confirmed by several studies, which measured a smaller CSA in standing versus supine positions in the IJVs [4, 15, 26–29]. However, for the reasons above, the flow rate was significantly different by comparing studies. To give an idea, even the same research group of Valdueza found in healthy people significant differences by comparing [24] with [29]. IJVs flow in supine position was 700 (270) versus 480 (136), respectively; VVs flow in sitting position was 210 (120) versus 67 (42), respectively.

Recently, Monti et al. proposed a cut-off value of ≤503.24 mL/min as a differential value between supine and sitting positions of cerebral venous blood outflow as a diagnostic criterion of MS. More than 70% of MS patients fulfilled the cut-off value versus 29% of healthy controls and 43% of patients with other neurological diseases. Even this study shows significant overlapping between controls and neurological diseases.

Since the inflow through the internal carotid and vertebral arteries is not significantly different with the head at 0° and 90°, respectively, the question is where is the blood lacking in sitting position? It has been hypothesized that the rest of the blood might be stored in the collateral veins, because the jugular flow increases passing from the upper to the lower segment of the neck [26].

A lumped model, calculating the blood flowing in the collateral network, demonstrates that the increased flow, from the jaw to the chest, is due to the reentry of the collaterals into the main outflow route [25]. The novelty of the model is to measure the outflow over the individual inflow. From this point of view, only 1% of the brain inflow in normal controls was found in the collaterals, in the supine position, thus indicating that a very small amount of blood volume in physiology reenters through the collaterals into the caval system by skipping the IJV. On the contrary, a significantly higher rate of the inflow was found in the collaterals of people with CCSVI associated with MS, up to 61% of the inflow. This result offers an interpretative key of the study of Monti et al.

However, by comparing the flow rate measured in the IJV in normal people, all published studies readily show differences, as well as very high standard deviations [24–31]. This suggests a high variability in cerebral inflow/outflow rate among individuals on one side, but also the inaccuracy of the current ultrasonographic flow assessment, on the other.

## 4. Instrumental Errors in Venous Flow Ultrasound Assessment

The low reproducibility even among quantitative protocols for venous outflow measurement suggests technical problems linked with the current methodology. This is related to the method of measurement of the two variables which occurs for calculation of the flow rate. Since the TAV can be reliably measured exclusively from the Doppler spectrum registered in the longitudinal aspect of the vein, CSA is measured in this position using the cursor in the machine developed for diameter assessment from the B-mode image (Figure 1). It is assumed that the vein is circular and so the CSA is calculated by the application of the formula of the area of the circle:

(2) $$\begin{array}{c} CSA=R^{2}\times \Pi . \end{array}$$

**Figure 2:**
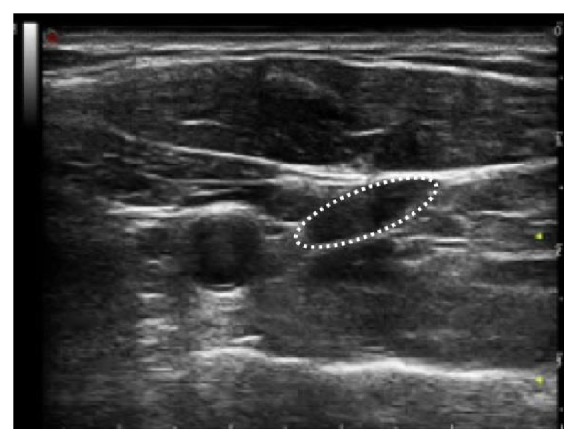
High resolution B-mode ultrasound imaging of the same case, in the transversal access of the neck, shows the elliptical shape of the internal jugular vein, outlined by the interrupted line.

This works very well when the ECD calculates the flow in an artery, because the shape is effectively a circle. But it works inaccurately when we need to measure the CSA of the internal jugular vein.  Figure 2 shows that the true shape of the jugular is elliptical and not circular. This means that there is a significant difference between the adjusted measure of the CSA and the true measure.

**Figure 3:**
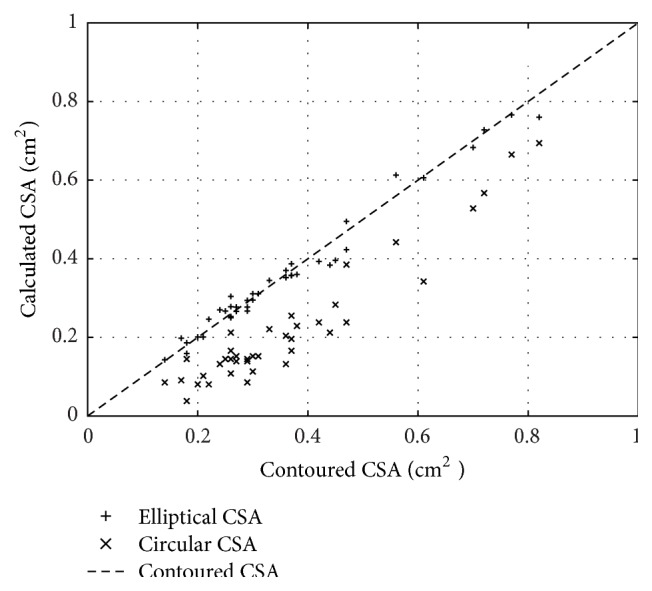
The elliptical (cross points) and circular CSA (× points) of the jugular vein of one subject are plotted as a function of the measured CSA (interrupted line), clearly showing the underestimation of the circular CSA. The automated measure of the circular CSA is based on the erroneous assumption of the circular shape of the jugular vein.

In a recent study, by the means of repeated countered measure of the elliptical internal jugular vein, a significant risk of flow rate underestimation with the assumption of a cylindrical venous shape has been demonstrated,. In Figure 3, the elliptical and circular CSA of the jugular of one subject are plotted as a function of the measured CSA, clearly showing the underestimation of the automated measure of CSA by the means of the current ultrasonographic equipment [30].

The second critical point of the current methodology is based on the assumption of just one constant measure of CSA over the time. On the contrary, the CSA of the jugular varies over the cardiac cycle, being a pulsatile large vein. It is known how CSA of the jugular significantly varies determining the so called phenomenon of the jugular venous pulse (JVP) [32, 33]. All the papers above reported the flow calculation by the means of a methodology based on single CSA value and TAV, despite the pulsed nature of the IJV flow, clearly visible at naked eye. A complete and correct analysis of the IJV blood flow would take into account the pulsatile nature of this vein; to neglect this point will comport an error whose magnitude has to be established.

Finally, the measure of TAV with the current ECD is very accurate if a laminar flow is present in the vein. In the terminal region, close to the valve and to the subclavian junction, all the investigators report the presence of turbulences, and the measure of flow is significantly different in this segment by comparing studies [24–29]. In this venous segment, an error of about 30% in measuring the flow rate due to the uncertain TAV assessment [25] has been estimated.

Since the region more susceptible to intraluminal obstacles is the valvular region, an uncertain flow rate assessment in this region potentially leads to errors in screening CCSVI.

## 5. The Jugular Venous Pulse 

In the previous paragraph it has been shown that the jugular vein is a pulsatile vessel. Consequently, the variation of the CSA over time significantly contributes to an inaccurate flow assessment when using just a single CSA value in measuring the flow. A complete and correct analysis of the IJV blood flow would take into account the pulsatile nature of this vein; to neglect this point will comport an error whose magnitude has to be established. The IJV pulsatility, the so-called jugular venous pulse (JVP), represents a corner stone of clinical medicine. But it is exactly the change in CSA over the cardiac cycle that we need for an accurate evaluation of the CSA.

JVP is defined as the movement of expansion of the jugular veins due to changes in pressure in the right atrium. It provides valuable information about cardiac hemodynamics and filling pressure [32], characteristic wave patterns pathognomic of cardiac diseases [33], and an indirect estimate of the central venous pressure (CVP). The JVP evaluation can be useful in managing many emergency conditions for guiding the fluid administration as well as in the diagnosis and/or prognosis of many heart [34] and lung diseases, notwithstanding it is often neglected by clinicians [32]. JVP is traditionally evaluated by a physical examination that visually analyzes the change in volume of the IJV, with variable inclination of the upper body and the sternal angle as the reference point [35]. However, it is considered a misunderstood and difficult physical technique [36] and the accuracy of the estimated CVP is no better than 50–60%. Besides, the real CVP is invasively measured through the cannulation of the venous system, thus not feasible as a routine approach. Several methods have been reported aiming to measure JVP and CVP either noninvasively or in a minimally invasive approach [37–40] but no one showed enough accuracy and precision [37], neither completeness of provided information for clinicians nor ease of use to enter in the routine clinical practice.

**Figure 4:**
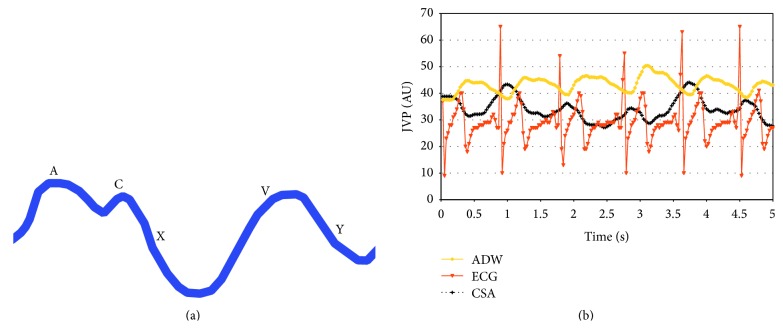
(a) The sequence of the 5 positive and negative waves composing the JVP, propagating the heart pressure upward the jugular vein. (b) Noninvasive ultrasonic assessment of JVP (black line) perfectly synchronized with the ECG trace (red line) and with the carotid artery distension wave (yellow line). The traces are derived by a high resolution B-mode video clip connected to an ECG. Their quantification allows a noninvasive assessment of the heart-brain axis.

JVP consists of three positive waves (A, C, and V) and two negative waves (descents) (X and Y). The A wave corresponds to atrial contraction and is synchronized with the P wave of the electrocardiogram (ECG). Descent X corresponds to lowering of the atrioventricular septum, interrupted by a small positive wave C in relation to closure of the tricuspid valve; the third wave V corresponds to cardiac systole and is followed by the Y descent, which corresponds to opening of the tricuspid valve (Figure 4) [32–36].

## 6. The Ultrasound Evaluation of JVP (US JVP)

The IJV is not only pulsatile, but one more important mechanical property of such vein is the distensibility. The latter was proven by the strong correlation between CSA and perimeter variation (*r* = 0.98) [41]. When the IJV is distended, the transmural pressure (i.e., the difference between the internal venous pressure and the atmospheric external pressure) and the CSA are correlated [42]; therefore, the time diagram of the IJV CSA reflects the JVP. B-mode ultrasound facilitates display of the cross section of the IJV and, therefore, measurement of the area and perimeter variation during the cardiac cycle [41].

In order to reliably derive the JVP through a sequence of CSA measurements of the jugular (US JVP), the subject is investigated with a high resolution real time B-mode video clip (about 24 frame∖sec), synchronized with an ECG trace. It has been recently demonstrated that US JVP represents a periodic signal that nicely reproduces the sequence of the five JVP waves, permitting a rapid, noninvasive assessment at bedside. Moreover, a semiautomatic tracing algorithm was developed to easily and rapidly derive US JVP in both research and clinical settings [41]. Since carotid and IJV are close with each other in the same anatomical region of the neck, it is now possible to derive from the same ultrasound video clip the ECG trace, the inflow signal [43], the arterial distension wave (ADW), and the outflow wave, that is, the JVP (Figure 4).

## 7. Perspectives

In the present issue of Behavioural Neurology, Sisini et al. propose a novel methodology based on the ultrasonographic assessment of the jugular venous pulse (US JVP) [44]. As reported above, they obtain ADW and JVP from high resolution ultrasound B-mode imaging, synchronized with the cardiac trace. The subsequent post analysis of such highly reproducible ultrasound imaging, by the means of the linear Womersley solution of the Navier-Stokes equations, permits taking a real picture with synchronism of the arterial and venous wave propagation with the heart pump. Results are very interesting and show the oscillating component of cerebral venous outflow. US JVP represents a novel system to assess the transmission of the cardiac waves upward the brain. In addition, the synchronization of the cardiac trace with the carotid artery distension gives us, noninvasively, a complete picture of the signalling which composes the so-called heart-brain axis. We actually ignore the impact on brain pathophysiology of the transmission of altered cardiac waves in consequence to extracranial venous pathology. But it seems as a promising field of research for the next years.

Finally, the work of Sisini et al., although intriguing, is really preliminary. Speculatively, we may think that the sequential assessment of IJV CSA is less prone to error with respect to the current methodology. Quite recently, the clinical applicability of this novel method of assessment has been successfully tested, measuring the reproducibility as well as estimating the instrumental error of such a novel assessment [45]. However, we need of a wider cohort of patients in order to assess the effectiveness of this technique in categorizing abnormal cerebral venous return in relationship with the cardiac circle. This is necessary in consequence of the inherent variability of the extracranial venous system among human subjects but also for a number of reasons linked with ultrasound, including pressure of the hand of the operator, respiratory movements, micromovements of the subject under investigation, and electronic noise. All these factors might again affect the accuracy of the measurement of cerebral venous return and warrant further studies in this direction.

## 8. The Future: Ultrasound Noninvasive Assessment of the Heart-Brain Axis

Historically, the concept of the heart-brain connection was born by the observation that a generalized autonomic storm, which occurs as a result of a life-threatening stressor, will have both sympathetic and parasympathetic effects. The effects of the autonomic nervous system on both organs were extensively studied in the past. Interestingly, ECG anomalies seen in the context of stroke did not represent ischemic heart disease but are merely a manifestation of autonomic dysregulation, possibly caused by a lesion that affected the cortical representation of the autonomic nervous system. Brodmann area 13 on the orbital surface of the frontal lobe and area 24 on the anterior cingulate gyrus are considered the cortical centers for cardiovascular control.

The heart-brain connection has many faces, but it can be divided into 3 major issues: the heart's effects on the brain (e.g., cardiac syncope or source of embolization), the brain's effects on the heart (e.g., neurogenic heart disease), and neurocardiac syndromes (e.g., Friedreich disease) [46].

More recently, the heart-brain axis seems to play a major role in the development of Alzheimer's disease (AD) and vascular dementia (VD), in consequence of the strong association of the increased risk to develop neurocognitive problems in patients with cardiovascular disease [47–50]. Epidemiology clearly shows the shared risk factors between AD-VD and cardiovascular risk factors. Noteworthy is that altered JVP is considered one of the major prognostic factors in cardiovascular diseases [32–34]. From this point of view, the novel opportunity to reliably assess US JVP could become a cornerstone in screening and prevention of neurocognitive disorders.
